# Prediabetes and the risk of breast cancer: a meta-analysis

**DOI:** 10.3389/fonc.2023.1238845

**Published:** 2023-09-18

**Authors:** Jing Lin, Rongzu Tu, Zhai’e Lu

**Affiliations:** ^1^ Health Management Center, Ningbo Women and Children’s Hospital, Ningbo, China; ^2^ Department of Internal Medicine, Ningbo Women and Children’s Hospital, Ningbo, China; ^3^ Department of Obstetrics, Ningbo Women and Children’s Hospital, Ningbo, China

**Keywords:** breast cancer, prediabetes, incidence, risk factor, meta-analysis

## Abstract

**Background:**

Diabetes has been related to a higher risk of breast cancer (BC) in women. However, it remains unknown whether the incidence of BC is increased in women with prediabetes. A systematic review and meta-analysis was therefore performed to evaluate the relationship between prediabetes and risk of BC.

**Methods:**

Observational studies with longitudinal follow-up relevant to the objective were found via searching Medline, Embase, Cochrane Library, and Web of Science. A fixed- or random-effects model was used to pool the results depending on heterogeneity.

**Results:**

Eight prospective cohort studies and two nest case-control studies were included. A total of 1069079 community women were involved, and 72136 (6.7%) of them had prediabetes at baseline. During a mean duration follow-up of 9.6 years, 9960 (0.93%) patients were diagnosed as BC. Pooled results with a fixed-effects model showed that women with prediabetes were not associated with a higher incidence of BC as compared to those with normoglycemia (risk ratio: 0.99, 95% confidence interval: 0.93 to 1.05, p = 0.72) with mild heterogeneity (p for Cochrane Q test = 0.42, I^2 =^ 3%). Subgroup analyses showed that study characteristics such as study design, menopausal status of the women, follow-up duration, diagnostic criteria for prediabetes, methods for validation of BC cases, and study quality scores did not significantly affect the results (p for subgroup analyses all > 0.05).

**Conclusion:**

Women with prediabetes may not be associated with an increased risk of BC as compared to women with normoglycemia.

## Introduction

Breast cancer (BC) is a highly prevalent malignancy among women worldwide, with approximately 1.4 million new diagnoses annually (1, 2). Established risk factors for BC include aging, family history of BC, and reproductive factors such as early menarche, late menopause, late age at first pregnancy, and low parity etc. (3). Early detection of BC is critical in preventing the disease, thus identifying populations at higher risk for its development is imperative (4).

Accumulating evidence suggests that hyperglycemia may have an adverse effect on BC incidence and prognosis (5, 6). A recent meta-analysis with 30 studies showed that patient with type 2 diabetes (T2D) were more likely to be diagnosed with BC as compared to those without T2D (7). Moreover, preexisting T2D has been also suggested to be a risk factor of poor survival of patients with BC (8). In the realm of glycemic metabolism research, the notion of prediabetes has emerged in recent decades as a means of characterizing a state of intermediate hyperglycemia that falls between normoglycemia and diabetes (9). Prediabetes is clinically defined by the presence of impaired glucose tolerance (IGT), impaired fasting glucose (IFG), and mildly elevated glycated hemoglobin (HbA1c) (10). As per established guidelines, IGT is diagnosed when plasma glucose concentrations range from 7.8-11.0 mmol/L after a 2-hour testing period of an oral glucose tolerance test. The definition of IFG is contingent upon the adoption of either the World Health Organization (WHO) or the 2003 American Diabetes Association (ADA) guideline definition, which respectively stipulate fasting plasma glucose (FPG) range of 6.1 to 6.9 mmol/L and 5.6 to 6.9 mmol/L (11). Furthermore, the American Diabetes Association (ADA) and the National Institute for Health and Care Excellence (NICE) also have classified HbA1c levels of 5.7–6.4% or 6.0–6.4% as prediabetic (12, 13). Prior researches have established a correlation between prediabetes and an elevated likelihood of experiencing cardiovascular events (14, 15), akin to the association observed with diabetes. However, the potential connection between prediabetes and an augmented risk of BC remains uncertain. Consequently, in the present study, a systematic review and meta-analysis was carried out to elucidate the relationship between prediabetes and the incidence of BC in female adult population.

## Methods

The PRISMA 2020 (16, 17) statement and Cochrane Handbook (18) were followed in this systematic review and meta-analysis.

### Database search

In order to identify studies that met the meta-analysis’ objectives, the following terms were combined (1): “prediabetes” OR “pre-diabetes” OR “prediabetic” OR “pre-diabetic” OR “prediabetic state” OR “borderline diabetes” OR “impaired fasting glucose” OR “impaired glucose tolerance” OR “IFG” OR “IGT” OR “fasting glucose” OR “HbA1c” (2); “breast”; and (3) “neoplasms” OR “carcinoma” OR “cancer” OR “tumor” OR “malignancy”. In the search, the dates of databases creation and the date of last search (April 12, 2023) were taken into consideration. Our selection criteria were limited to studies conducted on humans and published in English as full-length papers. Additionally, we manually checked the references of the related original and review articles to identify the original studies that were not included.

### Study identification

The PICOS criteria were followed in determining study selection criteria.

(1) P (Participants): Women without a known diagnosis of cancer at baseline.
(2) I (Intervention): Women with prediabetes at baseline. The diagnosis of prediabetes was in accordance with the criteria used in the original studies.
(3) C (Control): Women with normoglycemia at baseline.
(4) O (Outcome): The incidence of BC during follow-up durations, compared between women with prediabetes and women with normoglycemia.
(5) S (Study design): Observational studies that follow patients over time, including cohort studies, *post-hoc* analyses of clinical trials, and nested case-control studies.

Reviews, meta-analyses, editorials, studies enrolling patients with known cancer at baseline, studies without longitudinal follow-up, studies did not investigate prediabetes as exposure, or studies with no relevant outcomes were excluded.

### Study quality assessment and data extraction

For the purpose of assessing the study quality, the Newcastle–Ottawa Scale (NOS) (19) was used, which was composite of three domains involving defining groups of the study, comparing groups between them, and validating outcomes. The NOS incorporates nine criteria, and each study receives one point if it meets a specific criterion. As detailed above, two authors conducted electronic database searches, extracted study data independently, and assessed study quality independently. Disagreements between the two authors should be discussed in order to resolve them. The data collected were (1): study information (authors, countries, publication year, and study design) (2); sources and sample sizes of the included female population, and their mean ages (3); diagnostic criteria for prediabetes and numbers of participants with prediabetes at baseline (4); follow-up durations, number of women who were diagnosed as BC during follow-up, and methods for validating the outcomes; and (5) variables included in the multivariate regression analysis which was used for the analysis of the association between prediabetes and risks of BC.

### Statistical methods

Risk ratios (RRs) and 95% confidence intervals (CIs) were used to assess the association between prediabetes and risk of BC. For variance stabilization and normalization, we performed a logarithmical transformation followed by a calculation of the RRs and standard errors (SE) (18). An evaluation of heterogeneity was conducted using the Cochrane Q test and an I^2^ statistic (20). If I^2^ > 50%, heterogeneity was considered significant. A fixed-effects model was used to pool the results if heterogeneity among the included studies was not significant; otherwise, a random-effects model was used (18). Sensitivity analysis by excluding one dataset at a time was used to examine the stability of the finding. Subgroup analysis was carried out to evaluate whether the results were significantly affected by predefined study characteristics, such as study design, menopausal status of the women, follow-up duration, diagnostic criteria for prediabetes, methods for validation of BC cases, and study quality scores. In order to reflect publication bias, funnel plots were constructed and symmetry was examined visually. In addition, publication bias was simultaneously evaluated using Egger’s regression asymmetry test (21). The RevMan (Version 5.1; Cochrane Collaboration, Oxford, UK) and Stata (version 12.0; Stata Corporation, College Station, TX) software were employed for the statistical analyses.

## Results

### Database search results

**Figure 1:**
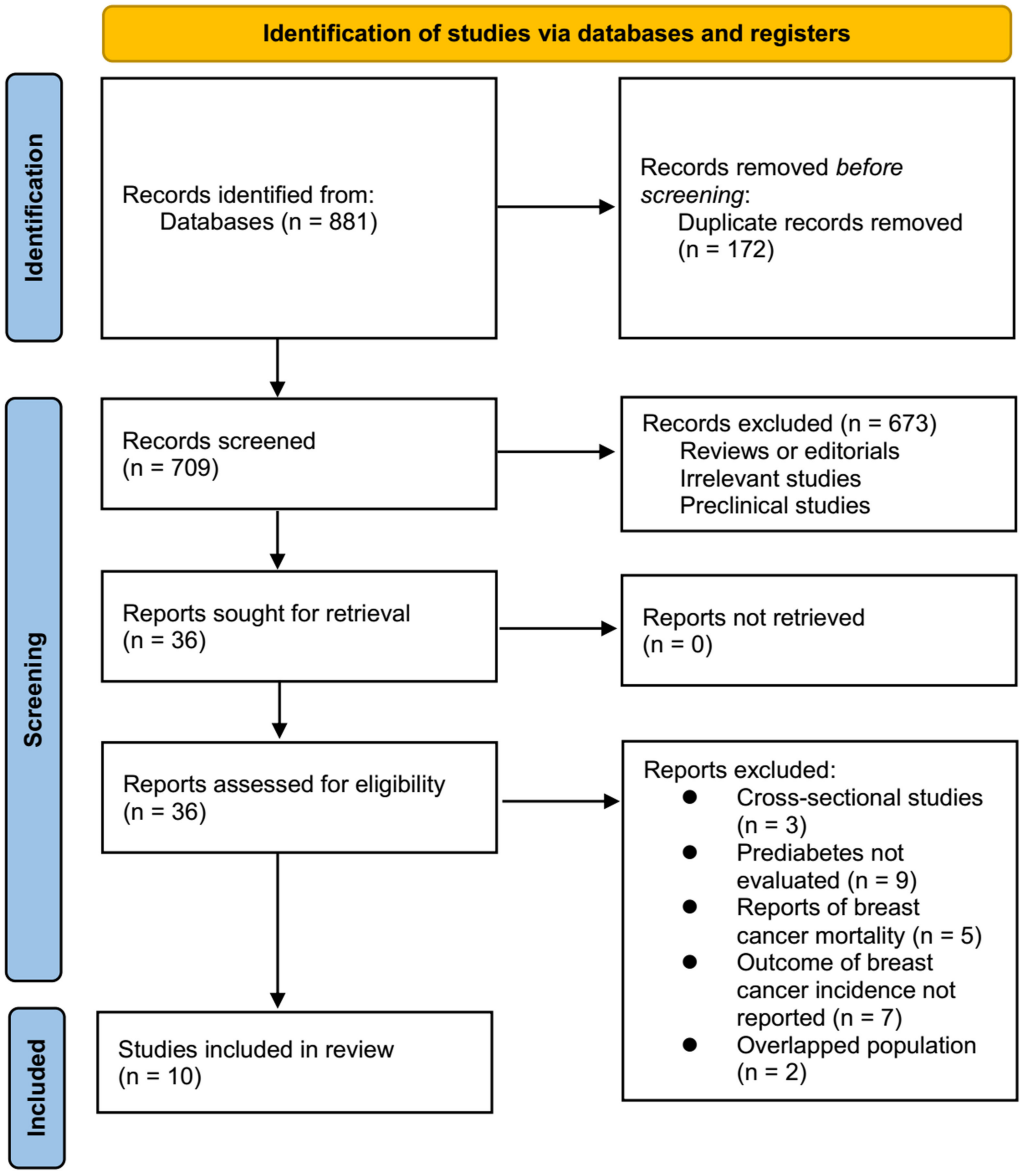
Flowchart of database search and study inclusion.

An overview of the database search process is shown in **Figure 1**. As a result of the initial literature search, 881 articles were found; after excluding duplications, 709 articles remained. As a result of screening the titles and abstracts, an additional 673 studies were excluded from the meta-analysis. A full-text review was conducted on the remaining 36 studies, of which 26 were further excluded for the reasons listed in **Figure 1**. As a final step, ten observational studies (22–31) were used for this meta-analysis.

### Characteristics of the included studies

Characteristics of the included studies are displayed in **Table 1**. Overall, eight prospective cohort studies (22–29) and two nest case-control studies (30, 31) were included. These studies were published between 2005 and 2022, and performed in Korea, Austria, the United States, Japan, Sweden, Canada, and the United Kingdom. A total of 1,069,079 community-derived women were included, with the mean ages of 37 to 65 years. Prediabetes was defined as IFG in five studies (22, 23, 25, 26, 28), as IFG and/or IGT in one study (24), and as HbA1c of 5.7~6.4% in four studies (27, 29–31). Accordingly, 72136 (6.7%) of the included participants had prediabetes at baseline. The mean follow-up durations were 6 to 37 years in the studies. During a mean duration follow-up of 9.6 years, 9960 (0.93%) patients were diagnosed as BC. Validation of BC was evidenced via national cancer registries in five studies (22–24, 26, 29), and via medical records in the other five studies (25, 27, 28, 30, 31). Variables such as age, body mass index, smoking, and alcohol drinking were adjusted in the multivariate regression models when the association between prediabetes and the risk of BC was analyzed in each study. A good quality study was indicated by a NOS range of eight to nine stars (**Table 2**).

**Table 1:** Characteristics of the included studies.

Study	Country	Design	Participants	Sample size	Mean age (years)	Diagnosis of PreDM	No. of PreDM	Follow-up duration (years)	Number of BC cases during follow-up	Validation of outcome	Variables adjusted
Jee 2005 (22)	Korea	PC	Community women aged 30 to 95 years	468615	49.6	IFG	22578	10	293	National cancer registry	Age, smoking, and alcohol use
Rapp 2006 (23)	Austria	PC	Community women	77228	43	IFG	3320	8.4	872	National cancer registry	Age, smoking, and alcohol use
Kabat 2009 (25)	USA	PC	Community women aged 50 to 79 years	4888	62.6	IFG	1277	8	165	Medical records	Age, ethnicity, BMI, oral contraceptive use, hormone therapy, alcohol intake, family history of BC, physical activity, energy intake, and smoking
Inoue 2009 (24)	Japan	PC	Community women aged 40 to 69 years	18176	55.5	IFG and/or IGT	2166	10.2	120	National cancer registry	Age, study center, smoking, alcohol drinking, and TC
Lambe 2011 (26)	Sweden	PC	Community women aged 25 years or older	230737	46.6	IFG	6843	11.7	6070	National cancer registry	Age, parity and age at first livebirth
Joshu 2012 (27)	USA	PC	Community women aged from 45 to 64 years	7003	56.2	HbA1c 5.7~6.4%	1509	15	379	Medical records	Age, race, study center, BMI, age at menopause, age at first livebirth, family history of BC, number of sisters, alcohol intake, and smoking
Parekh 2013 (28)	USA	PC	Community women aged 20 years or older	2308	37.5	IFG	350	37	217	Medical records	Age, alcohol, smoking, and BMI
Price 2020 (30)	Canada	NCC	Community women	591	65.1	HbA1c 5.7~6.4%	198	6	195	Medical records	Age, total physical activity, smoking status, chronic disease history, family history of BC, menopausal status and standing height
Peila 2020 (29)	UK	PC	Community women aged from 40 to 69 years	257044	56.3	HbA1c 5.7~6.4%	33495	7.1	761	National cancer registry	Age, education, non-white race, smoking status, alcohol intake, BMI, physical activity, family history of BC, number of live births, history of benign breast disease, use of contraceptive pills, and history of mammogram screening
Campbell 2022 (31)	USA	NCC	Community women	2489	NR	HbA1c 5.7~6.4%	400	7.5	888	Medical records	Age, sex, smoking, BMI, physical activity, alcohol, time since last ate at blood draw, and HRT

PreDM, prediabetes; BC, breast cancer; PC, prospective cohort; NCC, nested case-control; IFG, impaired fasting glucose; IGT, impaired glucose tolerance; HbA1c, glycosylated hemoglobin; NR, not reported; BMI, body mass index; TC, total cholesterol; HRT, hormone replacement therapy.

**Table 2:** Study quality evaluation via the Newcastle-Ottawa Scale.

Study	Representativeness of the exposed cohort	Selection of the non-exposed cohort	Ascertainment of exposure	Outcome not present at baseline	Control for age	Control for other confounding factors	Assessment of outcome	Enough long follow-up duration	Adequacy of follow-up of cohorts	Total
Jee 2005 (22)	1	1	1	1	1	0	1	1	1	8
Rapp 2006 (23)	1	1	1	1	1	0	1	1	1	8
Kabat 2009 (25)	1	1	1	1	1	1	1	1	1	9
Inoue 2009 (24)	1	1	1	1	1	0	1	1	1	8
Lambe 2011 (26)	1	1	1	1	1	0	1	1	1	8
Joshu 2012 (27)	1	1	1	1	1	1	1	1	1	9
Parekh 2013 (28)	1	1	1	1	1	0	1	1	1	8
Price 2020 (30)	0	1	1	1	1	1	1	1	1	8
Peila 2020 (29)	1	1	1	1	1	1	1	1	1	9
Campbell 2022 (31)	0	1	1	1	1	1	1	1	1	8

### Association between prediabetes and the incidence of BC

**Figure 2:**
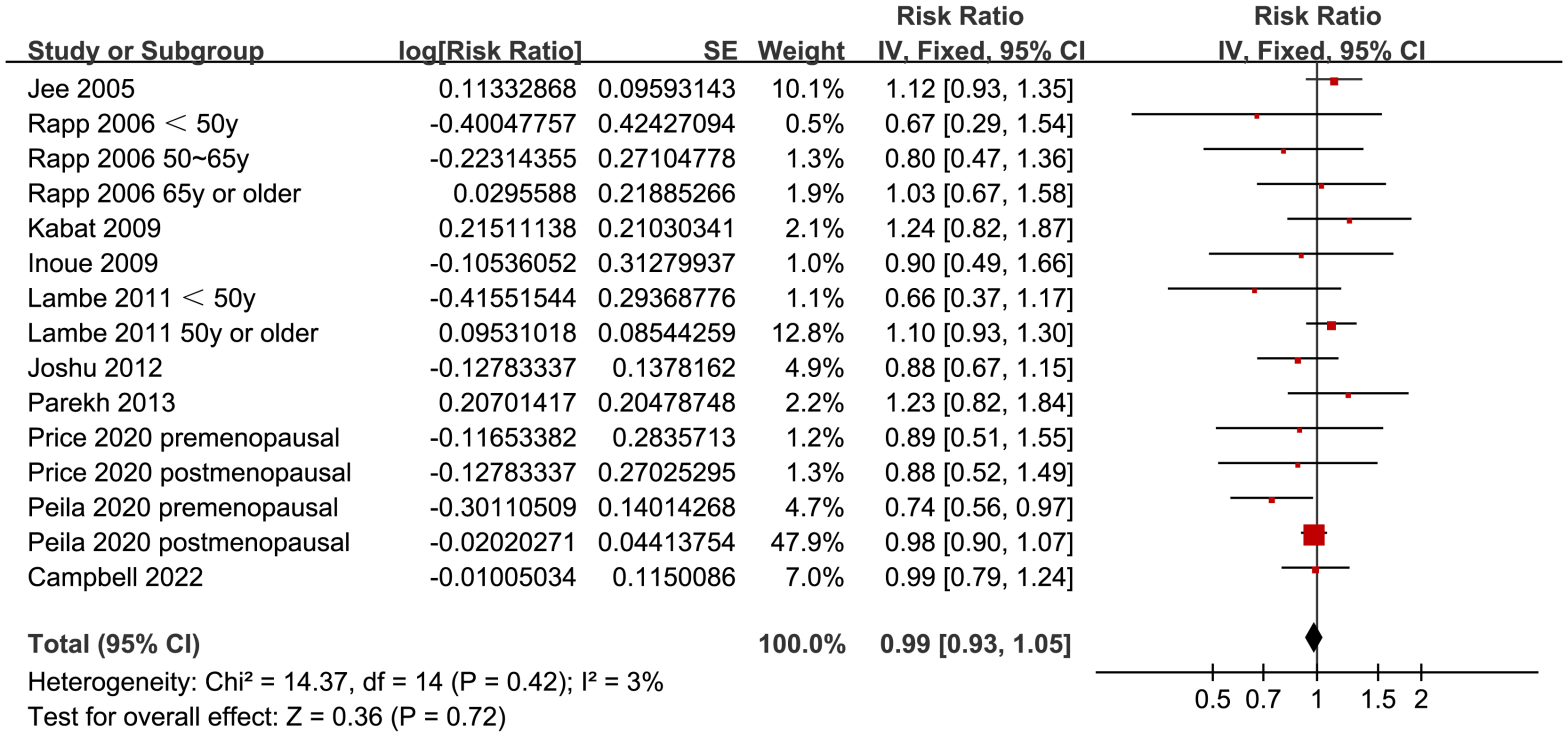
Forest plots for the meta-analysis of the association between prediabetes and the incidence of BC.

**Figure 3:**
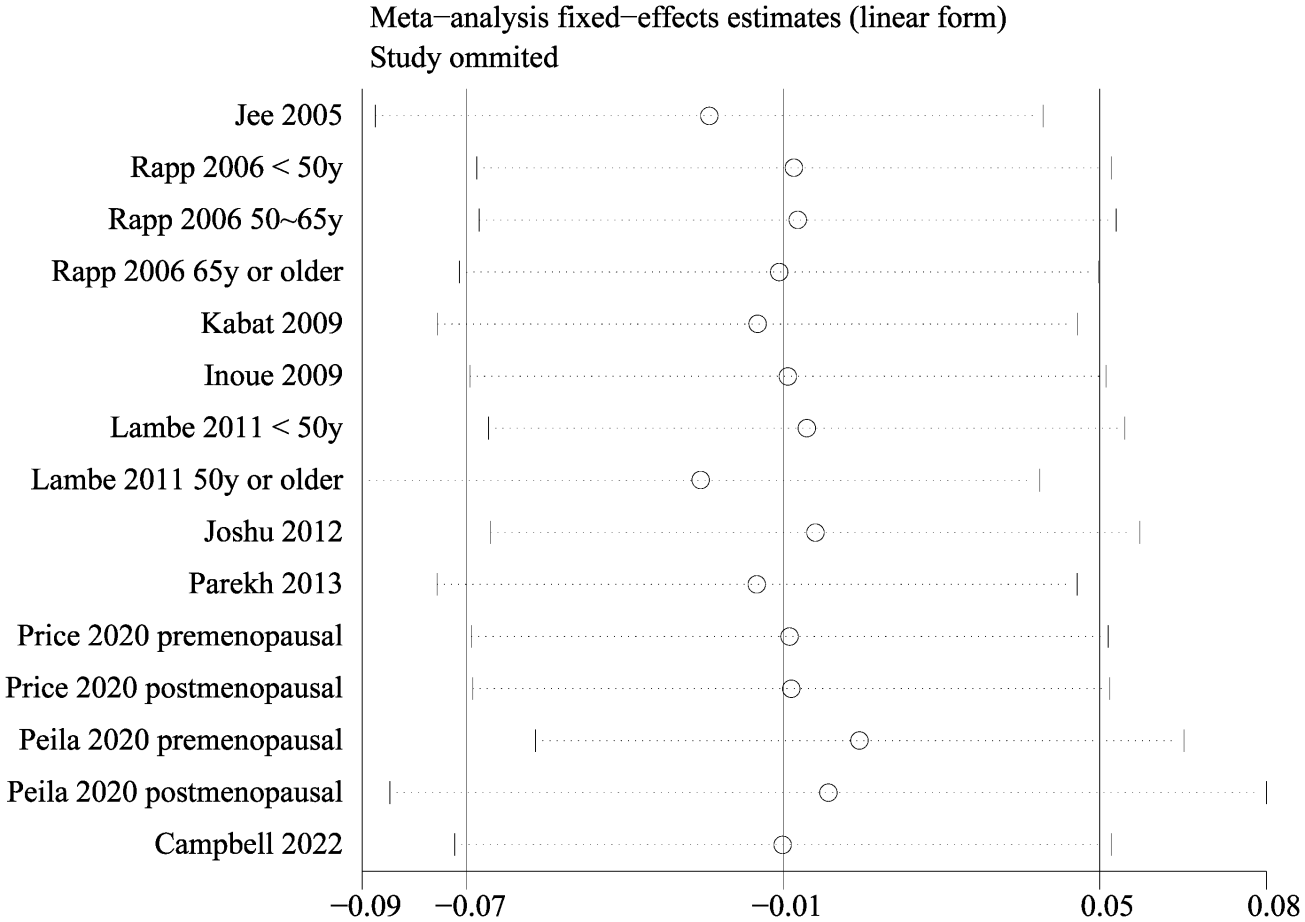
Results of sensitivity analysis by excluding one dataset at a time.

**Figure 4:**
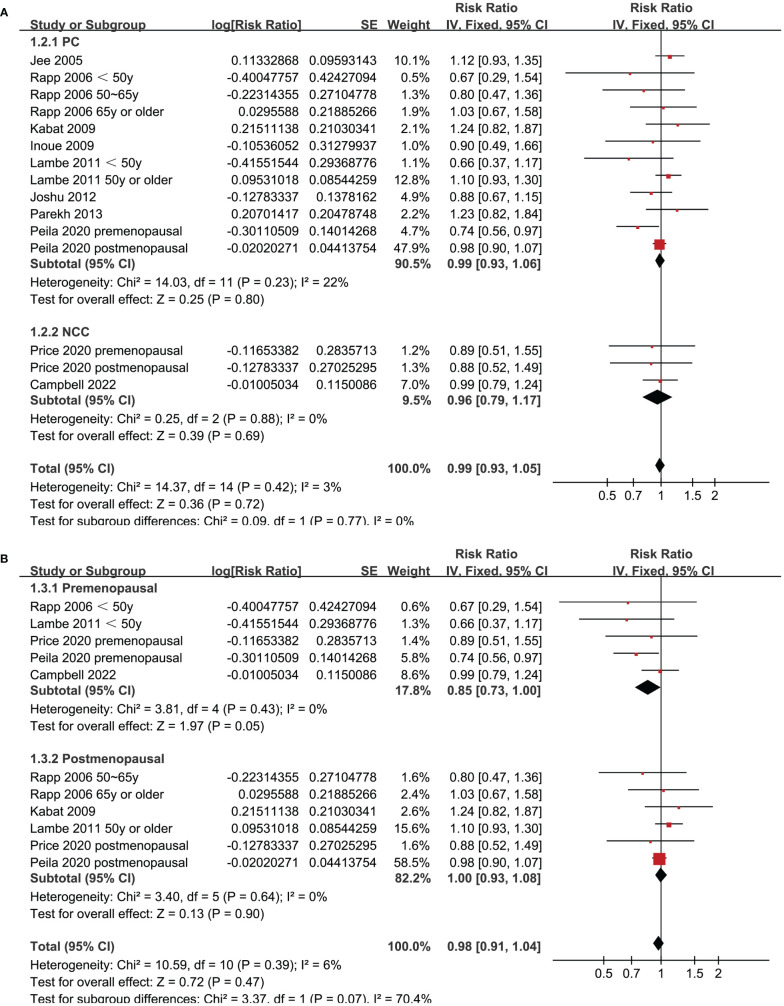
Forest plots for the subgroup analyses of the association between prediabetes and the incidence of BC. **(A)**, subgroup analyses according to study design; and **(B)**, subgroup analyses according to menopausal status of the women.

**Figure 5:**
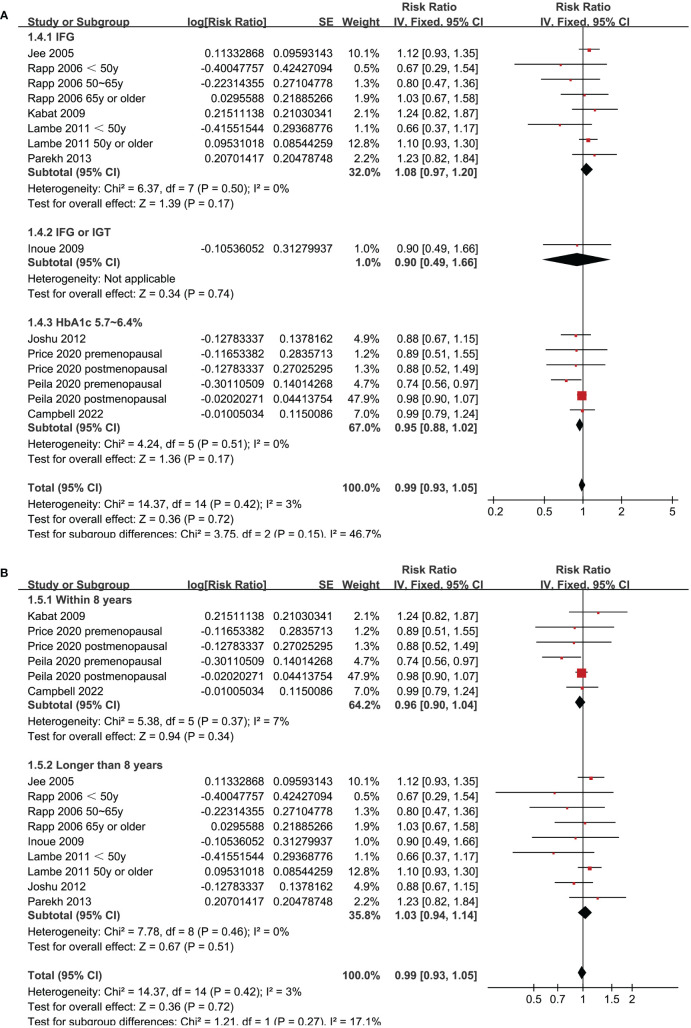
Forest plots for the subgroup analyses of the association between prediabetes and the incidence of BC. **(A)**, subgroup analyses according to definition of prediabetes; and **(B)**, subgroup analyses according to follow-up duration.

**Figure 6:**
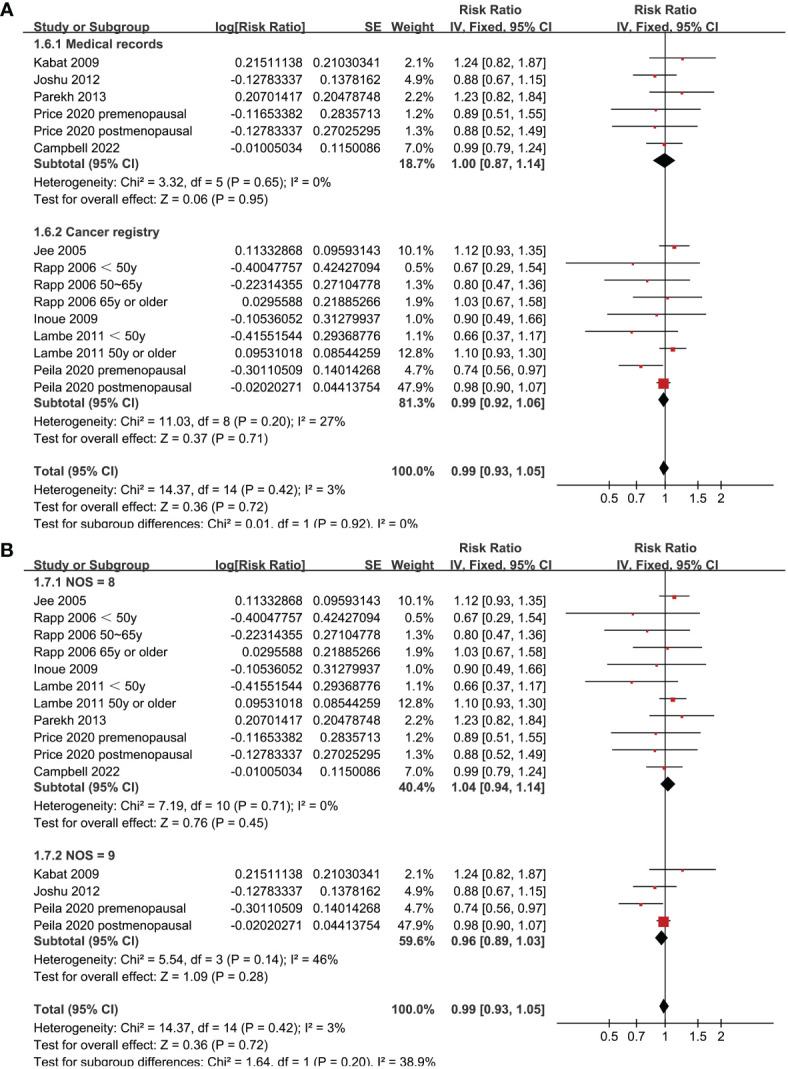
Forest plots for the subgroup analyses of the association between prediabetes and the incidence of BC. **(A)**, subgroup analyses according to methods for validation of BC; and **(B)**, subgroup analyses according to the study quality scores.

Since two of the included studies reported data according to the age of the included women, and another two studies according to the menopausal status of the included women separately, these datasets were included in the meta-analysis independently. Overall, 15 datasets from ten studies were available for the meta-analysis (22–31). Mild heterogeneity was observed among the included studies (p for Cochrane Q test = 0.42, I^2 =^ 3%). Pooled results with a fixed-effects model showed that women with prediabetes were not associated with a higher incidence of BC as compared to those with normoglycemia (RR: 0.99, 95% CI: 0.93 to 1.05, p = 0.72; **Figure 2**). Sensitivity analysis by excluding one dataset at a time showed similar results (RR: 0.97 to 1.01, p all > 0.05; **Figure 3**). Subgroup analyses showed that study characteristics such as study design (p for subgroup difference = 0.77, **Figure 4A**), menopausal status of the women (p for subgroup difference = 0.07, **Figure 4B**), diagnostic criteria for prediabetes (p for subgroup difference = 0.15, **Figure 5A**), follow-up duration (p for subgroup difference = 0.27, **Figure 5B**), methods for validation of BC cases (p for subgroup difference = 0.92, **Figure 6A**), and study quality scores (p for subgroup difference = 0.20, **Figure 6B**) did not significantly affect the results.

### Publication bias

**Figure 7:**
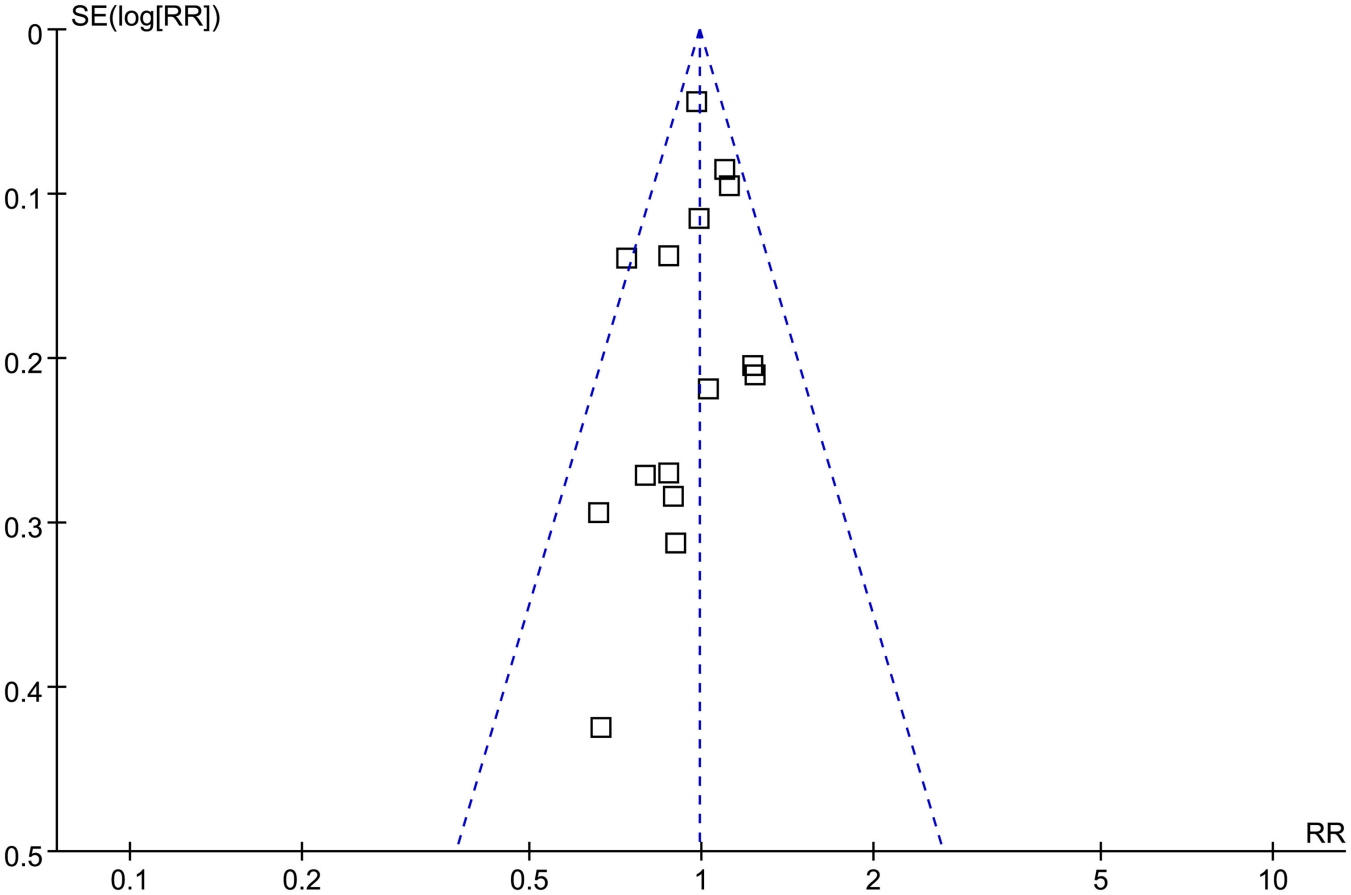
Funnel plots for the publication bias underlying the meta-analysis of the association between prediabetes and the incidence of BC.

**Figure 7** shows the funnel plots regarding the association between prediabetes and the incidence of BC. According to visual inspection, the plots are symmetrical, which suggests that high risk of publication bias is unlikely. Additionally, Egger’s regression tests indicated a low risk of publication bias (p = 0.52).

## Discussion

Based on this meta-analysis, women with prediabetes were shown to be not associated with an increased incidence of BC compared to controls with normoglycemia. Further sensitivity analyses by omitting one study at a time showed consistent results. Subsequent subgroup analyses showed that the results were not significantly by differences of study characteristics such as study design, menopausal status of the women, follow-up durations, definition of prediabetes, methods for the validation of BC cases, or study quality scores. As a result, these results indicate that prediabetes may not be a risk factor of BC in women.

Few previous meta-analyses have evaluated the association between prediabetes and risk of BC. Although an early meta-analysis incorporating the evidence from 16 cohort studies that found that overall prediabetes may be associated with an increased risk of cancer, subsequent subgroup analysis showed that the association may be site-specific according to different cancers (32). As for the subgroup analysis for BC, four cohort studies were included and the pooled results suggest that prediabetes may be associated with a higher risk of BC. However, besides studies reporting the incidence of BC, the authors also included a study that reported BC related mortality, which may confound the results of the meta-analysis (32). Some methodological strength should be noticed in the current systematic review and meta-analysis as compared to the previous one. For example, a comprehensive literature search in four widely used electronic databases was performed, which retrieved ten observational studies according to the aim of the meta-analysis. In addition, only studies reporting BC incidence were included, and studies reporting BC related mortality was excluded. This is important because the two outcomes are not always consistent because BC related mortality could also be influenced by therapeutic factors. Moreover, multivariate regression analyses were used among all of the included studies, and the results were independent of the potential confounding factors such as age, BMI, family history of BC, smoking and alcohol drinking etc. Finally, the stability and robustness of the finding was further confirmed by the consistent results in sensitivity and subgroup analyses. Collectively, results of the meta-analysis suggest that based on the findings from current epidemiological studies, prediabetes may not be a risk factor of BC in women.

Although it is shown in recent studies that the global burdens of premenopausal and postmenopausal BC have been both raising in recent decades, the risk factors of premenopausal and postmenopausal BC could be different (33). In this meta-analysis, a subgroup analysis according to the menopausal status of the women suggested that prediabetes presented a trend of lowered risk of BC in premenopausal women (RR: 0.85, 95% CI: 0.73 to 1.00, p = 0.05), but not in postmenopausal women (RR: 1.00, 95% CI: 0.93 to 1.08, p = 0.90). Although the between-group difference was not statistically significant (p = 0.07), it suggested that prediabetes might be a protective factor for BC in premenopausal women. A similar effect to prediabetes has been suggested by an early study evaluating the influence of metabolic syndrome (MetS) on the risk of BC (34). This meta-analysis included 17 follow-up studies showed that MetS was associated with an increased risk of BC in postmenopausal women, but significantly reduced breast cancer risk in premenopausal women (34). The underlying mechanisms are not clear at current stage (35). From our perspective, this might be explained by the potential role of insulin on ovarian androgen synthesis in premenopausal women. Prediabetes is characterized by hyperinsulinemia and insulin resistance. It is speculated that insulin’s stimulating effect on ovarian androgen synthesis may lead to ovarian hyperandrogenism (36), which in turn may reduce the risk of BC in premenopausal women (37). Large-scale prospective studies are needed to validate the influence of menopausal status on the association between prediabetes and BC, and determined to mechanisms involved.

In addition, subgroup analysis also suggested that the difference of the definition of prediabetes did not significantly affect the association between prediabetes and the risk of BC. Nevertheless, the results should be interpreted cautiously because none of the included studies defined prediabetes as IGT in these studies. In a recent meta-analysis, different definitions and diagnostic criteria were found to affect the association of prediabetes with diabetes risk (38). Therefore, further studies are needed to clarify if different definition and diagnostic criteria for prediabetes could affect the association between prediabetes and BC.

This study also has limitations. First, we could not determine whether the association was consistent across pathological types of BC. In addition, although all selected studies utilized multivariate regression analysis, residual confounding factors could not be excluded, such as the potential influences of dietary and other lifestyle factors that are related to the risk of BC. Finally, as mentioned previously, it remains to determine if difference in menopausal status and definition of prediabetes may affect the results of the meta-analysis.

## Conclusion

Based on the meta-analysis, prediabetes may not be associated with an increased incidence of BC in women. However, it remains to be investigated if the conclusion is universal in pre and postmenopausal women, and in prediabetes with different definitions and diagnostic criteria.

## Data availability statement

The original contributions presented in the study are included in the article/Supplementary Material. Further inquiries can be directed to the corresponding authors.

## Author contributions

JL designed the study. JL and RT performed literature review, study identification, quality evaluation, and data collection. JL and ZL performed statistical analysis and interpreted the results. JL drafted the manuscript. All authors revised the manuscript. All authors contributed to the article and approved the submitted version.
